# Preparation and Characterization of TPP-Chitosan Crosslinked Scaffolds for Tissue Engineering

**DOI:** 10.3390/ma13163577

**Published:** 2020-08-13

**Authors:** Ilaria Silvestro, Iolanda Francolini, Valerio Di Lisio, Andrea Martinelli, Loris Pietrelli, Anna Scotto d’Abusco, Andromeda Scoppio, Antonella Piozzi

**Affiliations:** 1 Department of Chemistry, Sapienza University of Rome, P.le A. Moro, 5, 00185 Rome, Italy; ilaria.silvestro@uniroma1.it (I.S.); iolanda.francolini@uniroma1.it (I.F.); valerio.dilisio@uniroma1.it (V.D.L.); andrea.martinelli@uniroma1.it (A.M.); loris.pietrelli@uniroma1.it (L.P.); andromeda.scoppio@gmail.com (A.S.); 2 Department of Biochemical Sciences, Sapienza University of Rome, P.le A. Moro, 5, 00185 Rome, Italy; anna.scottodabusco@uniroma1.it

**Keywords:** chitosan, scaffolds, tissue engineering, tripolyphosphate, bone

## Abstract

Scaffolds are three-dimensional porous structures that must have specific requirements to be applied in tissue engineering. Therefore, the study of factors affecting scaffold performance is of great importance. In this work, the optimal conditions for cross-linking preformed chitosan (CS) scaffolds by the tripolyphosphate polyanion (TPP) were investigated. The effect on scaffold physico-chemical properties of different concentrations of chitosan (1 and 2% *w*/*v*) and tripolyphosphate (1 and 2% *w*/*v*) as well as of cross-linking reaction times (2, 4, or 8 h) were studied. It was evidenced that a low CS concentration favored the formation of three-dimensional porous structures with a good pore interconnection while the use of more severe conditions in the cross-linking reaction (high TPP concentration and crosslinking reaction time) led to scaffolds with a suitable pore homogeneity, thermal stability, swelling behavior, and mechanical properties, but having a low pore interconnectivity. Preliminary biocompatibility tests showed a good osteoblasts’ viability when cultured on the scaffold obtained by CS 1%, TPP 1%, and an 8-h crosslinking time. These findings suggest how modulation of scaffold cross-linking conditions may permit to obtain chitosan scaffold with properly tuned morphological, mechanical and biological properties for application in the tissue regeneration field.

## 1. Introduction

The repair of injured tissues is an articulate process that could be favoured by application of new approaches accelerating the regeneration course. Indeed, current strategies, such as transplantation of organs, repairing with non-vital materials and devices, use of external devices to augment or substitute a non-functioning organ, do not provide optimal solutions because of several limitations related to these treatments. Tissue engineering foresees an interdisciplinary scientific approach that combines the most recent researches in materials science, technology, and life sciences. Generally, tissue engineering involves the presence of reparative cells, a structural template (scaffold), transport of nutrients and metabolites, and molecular and mechanical regulatory factors.

In the last decades, many researchers have been focused on development of scaffolding systems to be applied as supports to promote tissue repairing when self-regeneration is compromised. Scaffolds are three-dimensional systems equipped with a highly porous structure as ideal environment to favour cells attachment and proliferation [1]. Scaffolds must also provide an appropriate response to mechanical stress during tissue regeneration. In fact, a challenging goal of tissue engineering is the production of a device with properties resembling those of the damaged tissue such to assure a proper tissue regeneration [2]. Then, the main role of the scaffold is to reproduce the architecture of the natural environment (skin, muscles, cartilage, and bone) and to guarantee bioactivity favouring cell adhesion and new tissue growth [3]. Also, a high biocompatibility is necessary to avoid any possible foreign body reaction. Several materials such as aliphatic polyesters (e.g., polycaprolactone and polylactic and polyglycolic acid), polyester–urethanes and biopolymers, particularly polysaccharides, have been investigated as scaffolding materials for a variety of tissue engineering applications [4,5]. Polyesters, generally, present low surface energy (high hydrophobicity) and high crystallinity. These features adversely affect scaffold degradation rate and mechanical properties. In addition, polymer degradation provokes the release of acidic products causing a strong inflammatory response. As for polyester–urethanes, a wide range of physical and mechanical properties can be obtained by using properly monomers and synthesis techniques. In particular, the elasticity and stiffness of these polymers as well as degradation rate can be modulated by suitably choosing the soft segment. However, since the soft segment is a diol polyester, the issues concerning inflammatory response remain. In the latter decades, polysaccharides are receiving a great attention for the biomedical applications thank to their biocompatibility, bioactivity, low cost, and eco-friendly features, as they are derived from natural sources, but above all, to the presence of functional groups useful to improve their biological and mechanical properties [6,7,8]. Among polysaccharides, chitosan (CS) is the most employed thanks to its similarity to naturally occurring glycosaminoglycan and its excellent properties like biocompatibility, biodegradability, non-toxicity, and easy processability. Recently, CS combined with nanoparticles, fibres or blended with other polymers has achieved a predominant role as a candidate for biomedical applications [9,10,11]. CS is a linear copolymer composed of D-glucosamine and N-acetyl-D-glucosamine units connected by (1–4) glyosidic bonds obtained by N-deacetylation of chitin. In particular, a deacetylation degree (DD) of at least 60% must be present in the polymer to be named chitosan. The DD can influence the physico-chemical and biological properties of the polysaccharide, specially its hydrophilicity and biocompatibility. The degree of deacetylation usually ranges from 70% to 95%. The presence of amine functional groups in the CS backbone makes CS easy modifiable by both chemical reactions and physical interactions with a number of different molecules widening its applications [12,13]. CS modification also aims at enhancing its poor mechanical properties that often makes it unusable in some fields. Generally, the most common method to enhance CS mechanical resistance is polymer crosslinking by using chemical agents for the formation of covalent or ionic bonds among polymer chains. In particular, CS ionic crosslinking can be obtained by the formation of a physical network involving interactions between its NH_2_ groups (pKa 6.5), positively charged in acid conditions, and selected anionic molecules such as citrate, sulphate, oxalate, and tripolyphosphate [14,15]. This approach is preferred with respect to chemical crosslinking because it is easy, fast, and avoids the use of eventually toxic substances such as glutaraldhehyde or genipin. Tripolyphosphate polyanion (TPP) is one of the most used anionic crosslinkers for CS and investigated for fabrication of microparticles, membranes, hydrogels, scaffolds, or composite systems for the biomedicine [16,17,18,19,20,21,22]. Moreover, since phosphate groups are considered important for bone mineralization, TPP is also widely employed as a crosslinker for development of biomimetic polymer systems for bone regeneration [16]. The crosslinking degree can strongly influence the homogeneity, porosity, hydrophilicity, as well as final mechanical properties of the scaffold. Therefore, an accurate management of the cross-linking phase is crucial to obtain systems with specific performances for their application in tissue engineering. The simple mixing of chitosan and TPP solutions is not considered a suitable strategy to regulate the cross-linking reaction since an instantaneous gelation leading to a precipitate formation occurs. Therefore, different approaches have been employed to prepare CS-TPP systems with controlled crosslinking degrees like those based on the ionic strength-dependence of CS solubility in presence of NaCl or on the slow diffusion of TPP into CS solution to obtain homogeneous membranes [23,24]. Also, the crosslinking of preformed CS scaffolds or membranes by dipping in TPP solution has been studied [15,25]. In particular, this last approach, even if easy, was found to be strongly affected by experimental parameters used for the cross-linking reaction including the cross-linker concentration and pH conditions.

In this work, chitosan scaffolds obtained by freeze-drying and then cross-linked by immersion in a TPP solution were prepared. In order to study the effects of some experimental conditions on scaffold performance, two CS concentrations (1 and 2% *w*/*v*) were used for scaffold preparation while two TPP concentrations (1 and 2% *w*/*v*) and different reaction times (2, 4, and 8 h) were applied for scaffold cross-linking. The obtained systems were characterized by infrared spectroscopy, thermal and mechanical analysis as well as by swelling measurements. The morphological features, porosity and pore interconnection of the scaffolds were evaluated through scanning electron microscopy observations. Finally, to evaluate the potential application of the chitosan-based scaffolds in tissue engineering, preliminary studies of biocompatibility with osteoblast cells cultured on selected samples were performed.

## 2. Result and Discussions

**Figure 1:**
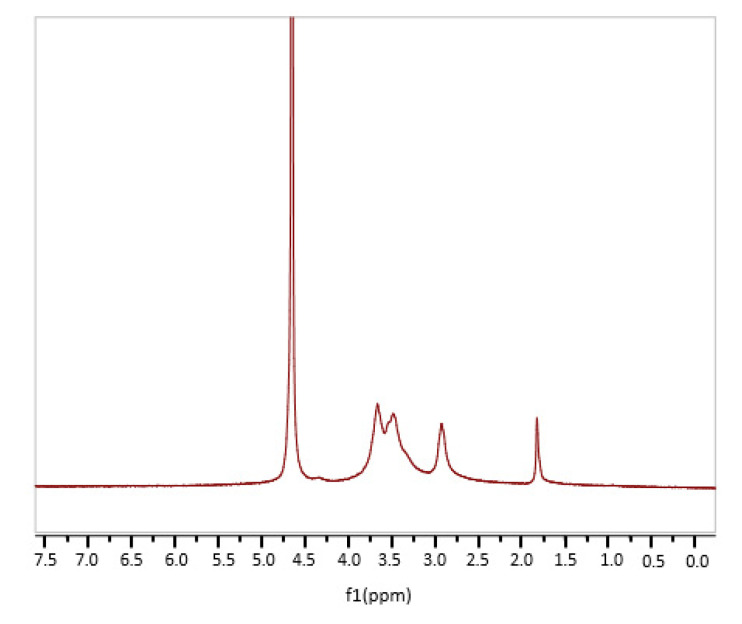
^1^H-NMR chitosan spectrum.

Scaffolds, as three-dimensional porous structures, should balance mechanical properties and biological function to promote a good regeneration process. Chitosan is a relevant candidate for tissue engineering because of its outstanding biological properties. As reported in the introduction section, the deacetylation degree of CS can strongly influence chitosan physical properties. In this work, a commercial CS with a nominal DD of 75–85% was used (see Materials and Methods section). ^1^HNMR analysis was carried out to determine the real CS DD. In the spectrum, reported in Figure 1, signals related to CH_3_ residue of the acetylated units at about 1.8 ppm and to H2, H3, H4, H5, and H6 protons of CS backbone in the range 2.5–4.7 ppm were present. In that range, the signal at 4.7 ppm of D_2_O was also visible. As reported in literature, by using integral intensities related to the peak of the three protons of acetyl group and those of the peaks of the CS backbone (H2–H6) a DD of about 80% was found [26].

Beside deacetylation degree, also molecular weight can affect the properties of the polysaccharide, particularly the mechanical ones. For applications in tissue engineering high molecular weight and high DD seem to be more suitable. As the manufacturer (Sigma Aldrich) did not supply the CS molecular weight, viscosity measurements were performed to determine it. The intrinsic viscosity was evaluated by using CS dissolved in aqueous solutions of acetic acid and sodium acetate at 25 °C. The viscosity average molecular weight resulted to be 280 kDA and was calculated by using the Mark–Houwink–Sakurada equation, η = K·M^α^, with K = 1.57 × 10^−4^ (dm^3^/g) and α = 0.79 [27].

**Figure 2:**
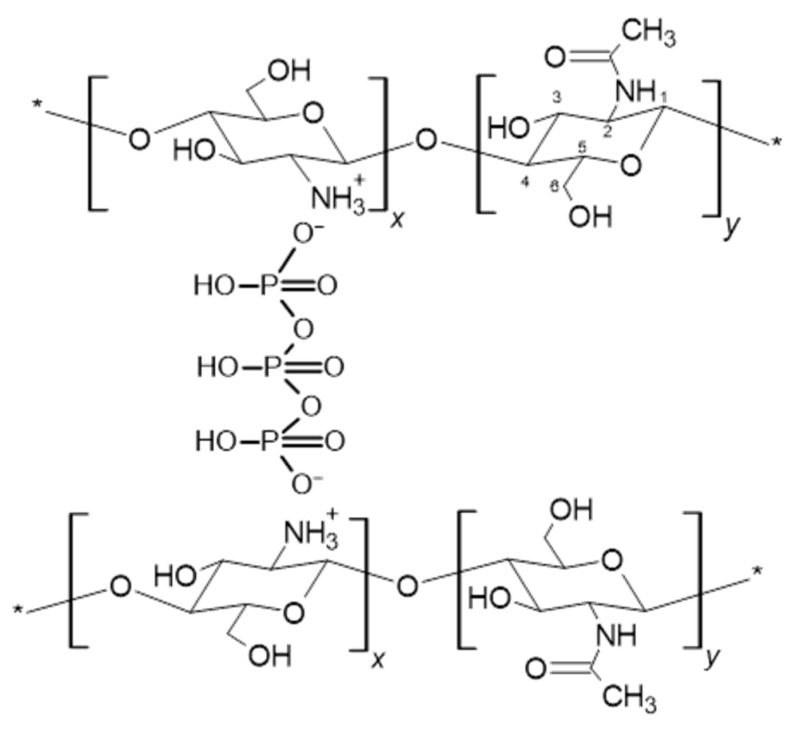
Ionic interactions of CS with tripolyphosphate polyanion (TPP).

However, high hydrophilicity (high DD value) makes this polysaccharide poorly mechanical stable in the biological environment, which may compromise its ability to substitute, even if temporarily, a damaged tissue. In this framework, many investigations have been focused on stabilization of CS structures for application in tissue engineering by either chemical or physical crosslinking. Specifically, CS physical crosslinking is usually carried out by interaction of the positively charged CS amino groups with negatively charged molecules thus obtaining a stable ionic network. In this work, chitosan scaffolds were prepared by freezing–drying and then crosslinked with TPP to improve scaffold stability and mechanical properties by establishment of CS/TPP ionic interactions. Indeed, at pH of the TPP solution (pH 9.1), OH^−^ and phosphoric ions, both present in the solution, can react with cationic groups of chitosan (NH3^+^), formed by the polymer dissolution in acidic condition (Figure 2). The OH^−^ groups are responsible for CS deprotonation, reaction that competes with the TPP ionic crosslinking one [28]. Different experimental conditions were studied to tune scaffold performance (Table 1). Specifically, the effect on scaffold physico-chemical properties of two CS and TPP concentrations as well as of different times of the cross-linking reaction were investigated. As for this latter parameter, since no significant effect was found by increasing the crosslinking time from 2 to 4 h, only the samples obtained at 2 and 8 h were characterized.

### 2.1. Infrared Spectroscopy Analysis

To obtain information about the cross-linking reaction, FTIR-ATR (attenuated total reflection) spectra of the CS_TPP scaffolds were recorded and compared with those of the pristine crosslinker agent TPP and pure CS scaffold.

**Figure 3:**
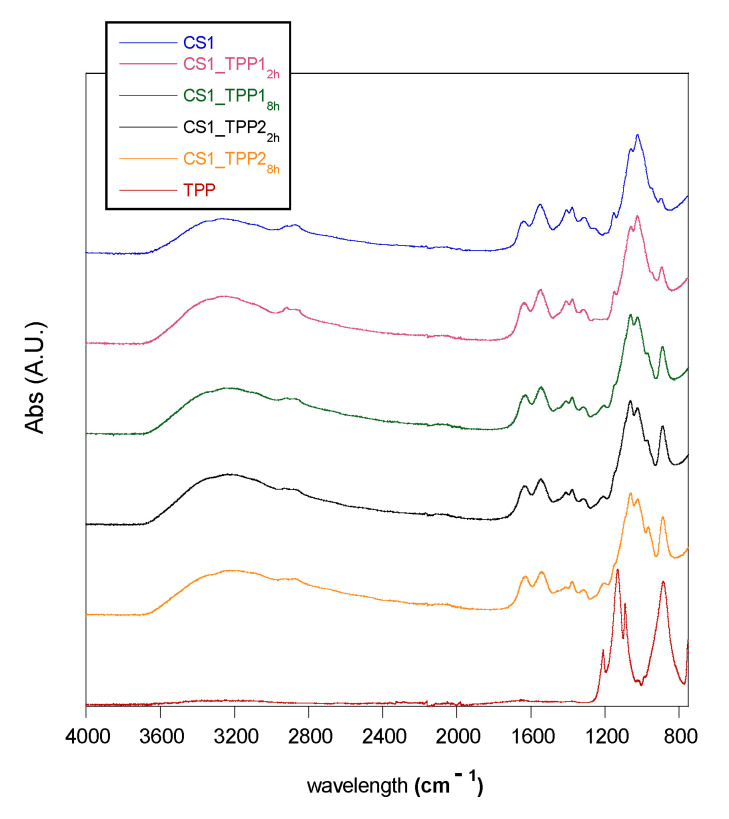
FTIR spectra of TPP, pristine chitosan (CS1), and crosslinked chitosan scaffolds (CS1_TPPY_z_).

In Figure 3, the spectra of CS1 and CS1_TPP, obtained employing two cross-linking times and TPP concentrations, were reported as an example. In the CS spectrum, the following characteristic bands can be observed: a broad absorption between 3500 and 3000 cm^−1^, corresponding to -OH and -NH stretching frequencies; the C-H stretching in the range 2920–2875 cm^−1^; the C = O stretching of acetylate groups (amide I) at 1650 cm^−1^; the N-H bending of primary amine at 1560 cm^−1^; absorptions due to the pyranose ring in the range 1150–1000 cm^−1^, particularly the C-O-C and C-O-H stretching at 895 cm^−1^ [29]. As for TPP, it is possible to observe the absorption bands at 1211 cm^−1^, relative to the P = O stretching, at 1130 cm^−1^ attributed to the symmetric and antisymmetric stretching of the -PO_2_ group, at 1090 cm^−1^ attributed to the symmetric and antisymmetric stretching of the PO_3_ group and at 881 cm^−1^ attributed to the antisymmetric stretching of the P-O-P bond [30,31].

By comparing the CS_TPP scaffold spectra with that of the pristine CS, several variations can be noted as a result of the cross-linking reaction. Indeed, in the spectra of the modified scaffolds, besides the peak at 881 cm^−1^ characteristic of TPP molecule, a new band at 1215 cm^−1^, related to the antisymmetric stretching vibrations of -PO_2_ groups of TPP ions, is present as an index of the formation of ionic interactions between CS and TPP [15,24,30]. That latter peak was not very pronounced in the spectrum of the CS1_TPP1_2h_ sample probably due to the low cross-linking degree (low reaction time and low TPP concentration used in the reaction), while it was more significant for the system obtained in more severe conditions of the cross-linking reaction (see sample CS1_TPP1_8h_). In order to have a semi-quantitative estimation of the cross-linking degree, the ratio between the intensity of the absorbance peak at 1560 cm^−1^ (related to the primary ammine of CS involved into the reaction) and that of the peak at 1650 cm^−1^ (related to the C = O groups of CS not involved into cross-linking process) was measured. In fact, due to the interaction between NH_3_^+^ groups and TPP, the intensity of the peak at 1560 cm^−1^ should decrease. In Table 2, the A_NH2_/A_C = O_ ratio for the CS_TPP samples and the pristine CS is reported. It can be seen as, regardless of the used CS concentration, this ratio decreases with the increasing of TPP concentration as well as with the reaction time confirming the influence of these parameters on the cross-linking reaction. A further confirm was given by the values of the absorbance ratio between the intensity of the peak at 881 cm^−1^ of TPP and that to 1650 cm^−1^ of CS (A_P-O-P_/A_C = O_). Indeed, an evident increase in polymer crosslinking with TPP concentration and reaction time increasing was observed (Table 2).

### 2.2. Thermogravimetric Analysis

**Figure 4:**
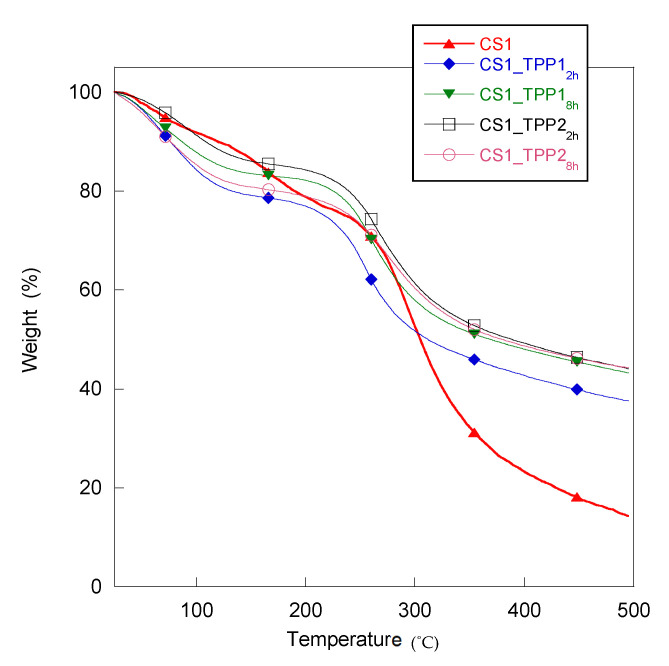
Thermogravimetric curves of CS1 and CS1_TPP scaffold obtained at different cross-linking conditions.

The study of thermal behaviour of the scaffolds can also provide an indication about the TPP-CS interactions within the ionic network. In fact, parameters as thermal stability and decomposition temperature are usually influenced by the cross-linking reactions. In Table 1, the values of the degradation temperature (Td) collected for the pristine CS and all the CS_TPP scaffolds are reported, while in Figure 4 the thermogravimetric curves of the CS1 and CS1_TPP samples are showed as an example. In all of the scaffold thermograms, the initial weight loss around 100–120 °C was attributed to evaporation of bonded water, while the weight variation observed in the range of 250–290 °C was related to the degradation of the polymer material. Generally, polymer cross-linking should increase material thermal stability. In our case, unexpectedly, a Td value lower than pristine CS for all of the cross-linked systems was evidenced. A similar result was obtained by Pati et al. by analysing the thermal behaviour of chitosan-TPP fibres compared to chitosan alone [23] and attributed to a reduction of the material crystallinity. Therefore, also in our case, a disturbing action of TPP on CS crystallinity may be hypothesized.

As for the CS1_TPP scaffolds, Td values increased with increasing in the TPP concentration and cross-linking time suggesting a better TPP interaction with the CS matrix. Indeed, the high thermal stability observed for the system CS1_TPP2_8h_ (Td = 277 °C) can be attributed to the formation of a more crosslinked structure. As for the CS2_TPP scaffolds fabricated with CS 2% (*w*/*v*), the same Td trend was detected with the increase in the TPP concentration from 1% to 2% (*w*/*v*) but not with the increase in crosslinking time. Indeed, a Td decrease with the crosslinking time was observed at both TPP concentrations (see data Table 1). Probably, in this case, the Td value was affected by the decrease in CS crystallinity due to TPP-CS interactions more than by the increase in cross-linking degree.

### 2.3. Morphological Properties and Porosity Tests

**Figure 5:**
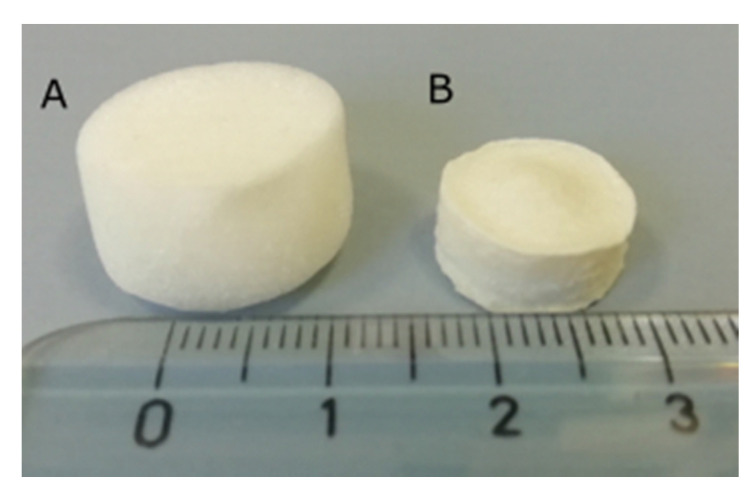
Illustration of pristine CS 1% scaffold (**A**) and cross-linked scaffold CS1_TPP1_2h_ (**B**).

The structure of the scaffold, particularly the pore distribution and size, is considered fundamental for its application in tissue engineering since may drive cell adhesion and migration. In Figure 5, an illustration of the pristine and crosslinked CS scaffolds is reported. Notably, after crosslinking a significant reduction in the scaffold size was observed.

**Figure 6:**
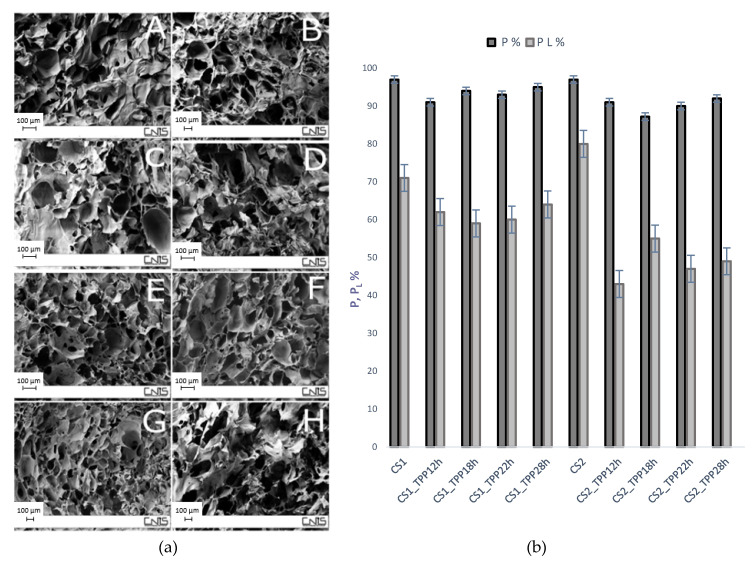
(**a**): Scanning Electron Microscopy (SEM) micrographs of the obtained scaffolds: pristine CS1 (**A**) and CS2 (**B**); CS1_TPP1_2h_ (**C**) and CS1_TPP1_8h_ (**D**); CS1_TPP2_2h_ (**E**) and CS1_TPP2_8h_ (**F**); and CS2_TPP2_2h_ (**G**) and CS2_TPP2_8h_ (**H**). (**b**): Total porosity (P%) and interconnected porosity (P_L_%) of the prepared systems.

Morphological analysis, performed by field emission scanning electron microscopy (FESEM), showed that the freezing-drying process is a good method to fabricate porous structures based on CS polymer (Figure 6(aA,aB)).

After reaction with TPP, such structures were slightly modified (Figure 6(aA–aH)). However, in general, the scaffolds showed a good morphological homogeneity between the external surface and the bulk structure, having similar mean pore size and distribution. The CS1_TPP1 scaffolds cross-linked for 2 and 8 h (Figure 6(aC,aD)) showed a variation of the pore morphology compared with the CS1_TPP2 scaffolds obtained with the same polymer concentration and reaction times (2 and 8 h) but different TPP concentration, precisely 2% (Figure 6(aE,aF)). In particular, a wide distribution of pore size (50–200 µm) was evident for the CS1_TPP1 samples contrarily to CS1_TPP2 scaffolds where a more homogeneity in the pore size and consequently a narrower pore size distribution (80–100 µm) were observed.

As far as the pore arrangement of the scaffolds obtained with CS 2% (*w*/*v*) is concerned, CS2_TPP2_2h_ (Figure 6(aG)) and CS2_TPP2_8h_ (Figure 6(aH)) had a more compact structure with slightly thicker pore walls compared to the CS1 scaffolds obtained with the same TPP concentration and reaction times (Figure 6(aE,aF)). Besides CS concentration, also a higher TPP concentration and a longer reaction time (sample CS2_TPP2_8h_ Figure 6(aH)) negatively affected the structure of the pores probably due to the strong interactions between the crosslinker and CS chains. This phenomenon was less pronounced for the CS2_TPP1_2h_ and CS2_TPP1_8h_ scaffolds crosslinked with TPP at 1% concentration for 2 or 8 h (data not shown). Overall, the best results in terms of scaffold morphology were found for the sample CS1_TPP2_8h_ (Figure 6(aF)).

An important parameter that affects the success of the tissue regeneration process is also the correspondence between the scaffold pore size and cell size. In this work, most of the samples showed an average pore size of about 100 µm that is generally accepted as the minimum pore size required for bone regeneration [32,33].

Scaffold porosity is also very important for cell adhesion and proliferation [34] and in this work was determined by using two different methods: the gravimetric method employed to determine the total porosity (P%) and the liquid displacement method (P_L_%) to evaluate the interconnectivity of the pores (see section Material and Methods). In Figure 6b, the obtained porosity data are reported as the average of four independent measurements. The values of P%, ranging from 87% to 97% for the all crosslinked systems, indicated the formation of the structures slightly denser than that of the pristine CS. Also the pore interconnectivity of the scaffolds (P_L_%) decreased after crosslinking underlining the key role of TPP in modifying scaffold structures. However, the P_L_% values seemed to be unaffected by the TPP concentration and reaction time.

More compact structures and with lower pore interconnection were instead obtained when the crosslinking process was performed on the scaffolds fabricated with CS2 compared to those obtained with CS1. Indeed, a porosity ≥60% was achieved in the CS1_TPP scaffolds, while a value ranging from 40 to 50% was found for the CS2_TPP scaffolds. Probably, at high CS concentrations, more NH_2_ groups were involved in the interaction with TPP molecules causing a partial collapse of the structure.

Results from the porosity determination confirmed that the CS1_TPP scaffolds possessed a structure better than CS2-TPP samples, with a good total porosity and interconnection degree suitable for their application in bone tissue engineering [33,35].

### 2.4. Water-Uptake Kinetics

**Figure 7:**
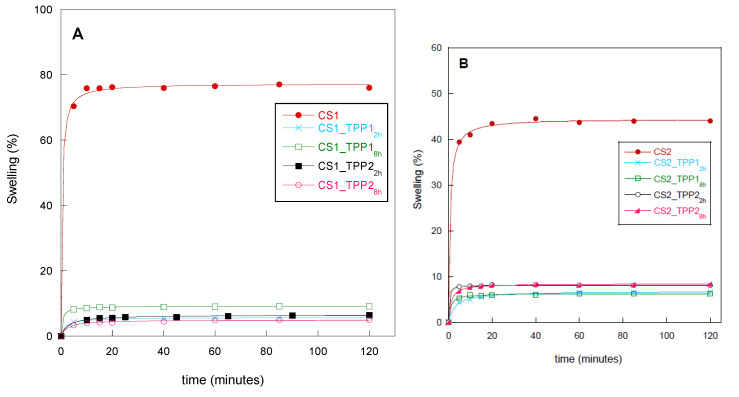
Swelling kinetics of the scaffolds obtained at different cross-linking conditions: (**A**) CS1 and CS1_TPP and (**B**) CS2 and CS2_TPP.

To verify the dimensional stability of the prepared scaffolds in an environment simulating physiological conditions, the scaffold swelling behaviour was evaluated in PBS buffer pH 7.4. The free ammine groups of CS make it a hydrophilic polymer with a great capacity to absorb water. The swelling kinetics was carried out by weighing the scaffolds at different times until the reaching of the equilibrium (constant weight). The pristine CS scaffold showed the highest affinity for water, reaching the maximum value of swelling degree (SD) in 5 min with a pronounced variation of its geometry (Figure 7). The maximum SD values depended on CS concentration, being 45% and 75% for CS1 and CS2, respectively (Figure 7A,B). Probably, higher CS concentration favoured the formation of a more compact scaffold structure (more entanglements among the chains) providing less access to water molecules. A similar behaviour was reported by J.E. Lee et al. who studied scaffolds fabricated with chitosan at 2 and 3% [36]. Furthermore, they found a decrease of the pore size of the structures related to the use of more concentrated solutions not evidenced in our case.

Generally, the scaffold cross-linking reaction should increase its stability in aqueous medium without drastically compromising the water adsorption capacity needed for cell penetration. According to the literature, a decrease in water uptake is considered as a confirmation of the ionic interactions between CS and TPP, being CS ammine groups less free to interact with water molecules [37]. Even though a strict relation between the swelling and cross-linking degree of the CS1 and CS2 scaffolds was not found, it was possible to observe that the TPP introduction caused a marked reduction of the water uptake compared to the pristine CS scaffolds (Figure 7A,B). The ability to absorb water remained unmodified for all of the samples in the tested period and no phenomena of dissolution was observed. This behaviour confirmed the good physical stability and integrity in aqueous media of the prepared scaffolds.

### 2.5. Mechanical Properties

**Figure 8:**
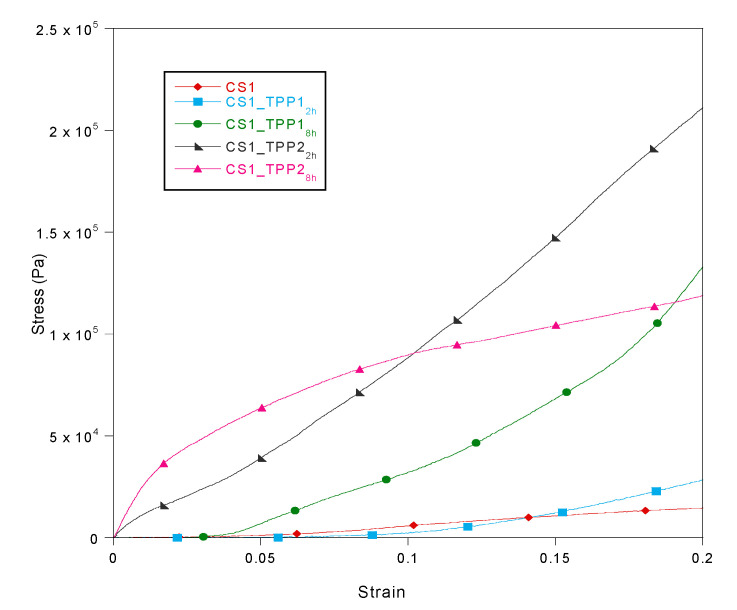
Stress–strain curves for the CS1 and CS1_TPP scaffolds.

The ability of the scaffold to match the mechanical properties of native tissue is strictly related to the success of the regeneration process. In fact, the scaffold should be able to resist to the many solicitations to which it would be submitted during the healing period as well as guarantee an optimum response to stress. In order to verify the mechanical resistance of the obtained structures, the compression modulus (CM) was collected for the pristine CS and CS_TPP scaffolds prepared under different conditions. The CM values were reported in Table 1 while the stress–strain curves of the CS1_TPP scaffolds were pictured in Figure 8, as an example. For determination of the CM value, the slope of the curve until 10% of deformation was considered. After that deformation, a collapse of the structure was observed causing an increase of the strength value due to the bulk effect of the polymer. Generally, the scaffolds obtained with a CS concentration of 2% (*w*/*v*) showed a greater resistance than those fabricated by using CS 1%. This behaviour is consistent with the fact that at high CS concentration more interactions among the chains occur with a consequent improvement of the scaffold mechanical strength. CS mechanical properties were improved by CS crosslinking with TPP. In particular, the increase in CM values was directly related to the TPP concentration (greater cross-linking degree), such finding in agreement with literature data [35,38]. A higher cross-linking time also positively affected the scaffold mechanical properties, confirming the importance of this parameter on the scaffold performances. Indeed, the highest CM value of 4.7 MPa was found for CS2_TPP2_8h_, obtained with more severe cross-linking conditions (2% TPP concentration and 8 h cross-linking time). It should be noticed that the scaffold mechanical properties are also related to the structure porosity. Even if a high porosity is suitable for a good interaction between the system and biologic environment, it could compromise the mechanical performance of the scaffold. Indeed, the improved mechanical behaviour of the CS2_TPP samples was presumably due to not only a greater crosslinking but also a less porosity (P_L_%). In conclusion, the better CM values were obtained for the samples CS1_TPP2_8h_, CS2_TPP2_2h_ and CS2_TPP2_8h_, which showed a modulus approaching the typical value of trabecular bone (ranging between 2 and 3000 MPa) [35,38].

### 2.6. Cell Viability Tests

**Figure 9:**
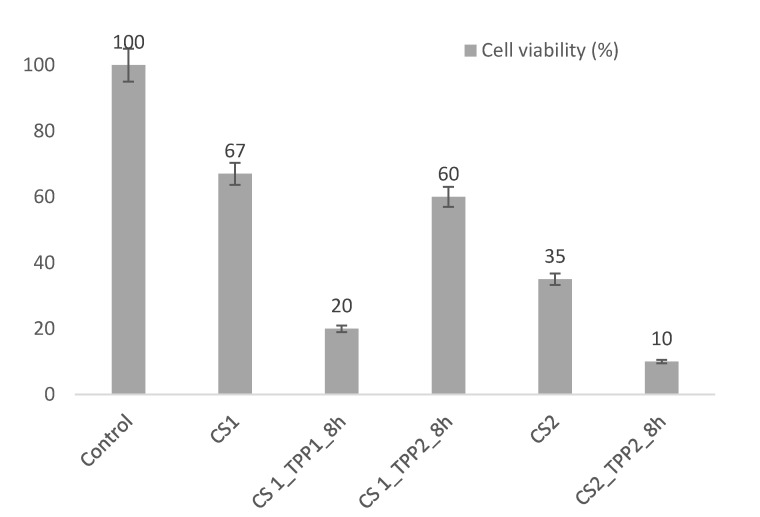
Cell viability (CV) of osteoblasts seeded on selected samples.

In order to assess the biocompatibility of the obtained scaffolds, a preliminary study on viability of osteoblast cells seeded on the CS structures were performed by using the MTS assay (see Materials and Methods section). For this test, the scaffolds obtained with 8-h crosslinking reaction time but different TPP and polymer concentration were selected. Indeed, in these CSX_TPPY_8h_ samples a good compromise between porosity and mechanical properties was reached. To test the effect of the TPP introduction on cell viability (CV), CS1 and CS2 scaffolds were used as controls. The results, reported in Figure 9, showed as CS1 possessed the highest biocompatibility, equal to 67% cell viability, differently from CS2 (35% CV). Probably, the more compact structure of the CS2 scaffold was responsible of the reduced viability of the osteoblast cells. The crosslinking reaction also led to a CV reduction likely caused by low pore homogeneity or interconnectivity. In fact, values of 20% and 10% CV for the CS1_TPP1_8h_ and CS2_TPP1_8h_ structures, respectively, were measured. In contrast, the CS1_TPP2_8h_ sample provided a good environment for the osteoblasts allowing this system to keep alive 60% of the seeded cells.

## 3. Materials and Methods

### 3.1. Viscosity and ^1^H-NMR Measurements

Chitosan (CS) with medium molecular weight, 200–800 cP and 75–85% deacetylation degree, was purchased by Sigma Aldrich, Darmstadt, Germany (animal origin, CAS number 9012-76-4). To determine the CS molecular weight, viscosity measurements were performed at 25 °C by using an automatic system SCHOTT GERÄTE Ubbelohde capillary viscosimeter equipped with a SCHOTT AVS 350 ViscoSystem and a LAUDA CD15 thermostatic bath. CS was dissolved in an aqueous solution of acetic acid and sodium acetate (CH_3_COOH 0.25 M/CH_3_COONa 0.25 M) (Sigma Aldrich, Darmastadt, Germany). All of solutions (concentration range from 0.01 to 0.1% *w*/*v*) were stirred for 1 h and then filtered with GF/D Whatman^®^ microfiber glass filter. ^1^H-NMR spectroscopy (XL 300, Varian, Palo Alto, CA, USA) was instead used to determine the chitosan deacetylation degree (DD). CS spectrum was performed employing a Varian XL 300 instrument and D_2_O as the solvent.

### 3.2. Preparation of CS_TPP Scaffolds

Chitosan solution was prepared by dissolving the polymer powder in an acid acetic solution (0.4% *v*/*v*) obtaining two different concentrations (1% and 2% *w*/*v*). The solution was stirred for 24 h at room temperature and then centrifuged at 3500 rpm for 10 min to remove air bubbles incorporated during the mixing process. Hence, 3 mL of each solution was poured in cylindrical plastic molds, frozen at −20 °C overnight and then freeze-dried for 24 h. The obtained CS scaffolds were immersed in a TPP (sodium tripolyphosphate, Alfa Aesar) solution at pH 9 for 2, 4, or 8 h, employing two different TPP concentrations (1 and 2% *w*/*v*). The crosslinking reaction was carried out at room temperature. In order to remove unreacted TPP, the scaffolds were washed in PBS buffer (pH 7.20) for several times and then frozen by immersion in liquid nitrogen and freeze-dried. Pristine chitosan scaffolds were also prepared as controls. Acronyms used for the scaffolds were: CSX for the pristine chitosan scaffold and CSX, PPY_z_ for the crosslinked samples, where X indicated CS concentration (1% or 2%), Y the crosslinker concentration (1% or 2%) and Z the crosslinking reaction time (2, 4, or 8 h).

### 3.3. Infrared Spectroscopy (FTIR)

The crosslinking reaction was evaluated by infrared spectroscopic analysis (FTIR). Spectra were acquired in attenuated total reflection (ATR) by a Nicolet 6700 (Thermo Fisher Scientific, Waltham, MA, USA) equipped with a Golden Gate single reflection diamond ATR accessory at a resolution of 2 cm^−1^ and co-adding 100 scans.

### 3.4. SEM Observation

The morphology and the pore size of the scaffolds were studied by field emission scanning electron microscopy (FESEM, AURIGA Carl Zeiss AG, Oberkochen, Germany). For analysis, the scaffolds were fractured by immersion in liquid nitrogen to observe their bulk structure. The scaffold external surface was also analysed. Both fractured and not-fractured surfaces were gold sputtered before observation. The pore size and regularity were obtained by micrograph observation of the sample bulk.

### 3.5. Determination of the Scaffold Porosity and Interconnection Degree

The gravimetric method was used to determine the total scaffold porosity (P%) by measuring apparent density of the scaffold (ρ_s_) compared with density of the pristine chitosan (ρ_c_), considered equal to 1.41 g/cm^3^. In order to determine ρ_s_, the weight and volume of the scaffolds were measured. The equations used for the determination of ρ_s_ and P% were:

(1) $$\varrho_{s}=\frac{m}{V}$$

(2) $$P\%=\left(1-\varrho_{s}/\varrho_{c}\right)\times 100$$

A liquid displacement method was used to evaluate how an ideal liquid could penetrate the pores to have information on pore interconnectivity. A scaffold with weight *W*_0_ and volume *V*_0_ was dipped in a volume *V*_0_ of ethanol for 30 min. Ethanol (density 0.806 g/cm^3^ at 20 °C) was chosen because it is a non-solvent for chitosan and does not provoke swelling phenomena. After that time, the scaffold was removed and weighted (*W*_1_). In this case, the interconnected porosity (*P_L_* %) was determined by employing the following equation [39]:

(3) $$P_{L}\;\%=\left(\frac{W_{1}-W_{0}}{\rho_{EtOH}\times V_{0}}\right)\times 100$$

### 3.6. Water-Uptake Kinetics of Scaffolds

The water uptake (*W*) of the scaffolds was determined by immersion of the weighted scaffolds (*W*_0_) in phosphate (PBS) buffer (pH 7.4) at room temperature and constant humidity. At different times, the scaffold has been collected, lightly dabbed on a filter paper to remove excess of solvent and weighted (*W_t_*). The test was carried out until saturation. Water uptake was defined as follows:

(4) $$W\left(\%\right)=\left(\frac{W_{t}-W_{0}}{W_{0}}\right)\times 100$$

Three parallel swelling experiments were performed for each sample and data were reported as average value ± standard deviation.

### 3.7. Thermal and Mechanical Characterization

Thermo-gravimetric analysis (TGA) was carried out employing a Mettler TG 50 thermobalance (Mettler Toledo, Columbus, OH, USA). Briefly, few milligrams of each sample were submitted to a temperature ramp with a heating rate of 10 °C·min^−1^ under N_2_ flow in the temperature range 25–500 °C. Then, the change in weight of the sample as a function of temperature was recorded.

Mechanical properties of the CS scaffolds were studied by tensile tests using an ISTRON 4502 instrument (Instron Inc., Norwood, MA, USA). For analysis the scaffolds with a cylindrical shape (diameter of 1.8cm and height of 1 cm for pristine CS while diameter of 1.3 cm and height of 0.5 cm for CSX_TPPY_z_) were placed between the two Instron flat plates and a constant deformation rate of 1 mm/min was set while a 10 N load cell was used. The compression modulus (CM) for the pure chitosan and cross-linked scaffolds was determined by the slope of the linear section of stress–stain curve in a range of deformation <10%. Three parallel experiments were performed for each sample and data were reported as average value± standard deviation.

### 3.8. Cell Viability

Cell viability was quantified after 7 days of incubation of osteoblast cells (Saos-2, ATCC^®^ HTB-85™, homo sapiens bone osteosarcoma) seeded on the CS structures by measuring the mitochondrial dehydrogenase activity using the dye 3-(4,5-dimethylthiazol-2-yl)-5-(3-carboxymethoxyphenyl)2-(4-sulfophenyl)-2H-tetrazolium (MTS) (Promega Corporation, Madison, WI, USA). Briefly, cells were seeded onto the CS scaffolds and after 7 days of incubation, 20 the MTS dye was added in the culture media and cells were cultured for 4 h to allow the formation of soluble formazan crystals by viable cells. Spectrophotometric absorbance was measured at 490 nm using a multi-plate reader Appliskan (Thermo Fisher, Waltham, MA, USA). Cells cultured in the absence of the CS_TPP matrices were taken as a control.

## 4. Conclusions

In order to fabricate CS_TPP structures suitable for tissue engineering applications, the effects of different CS and TPP concentrations (1 and 2% *w*/*v*) as well as crosslinking times (2, 4, and 8 h) on preformed scaffolds were investigated. FTIR analysis confirmed the polymer network formation evidencing an increase of the crosslinking degree with TPP concentration and reaction time increasing. Generally, the introduction of TPP into the CS preformed-scaffolds caused a decrease in the polymer crystallinity with reduction of the thermal stability of the systems. The CS crystallinity reduction was limited by increasing TPP concentration for crosslinking, as evidenced by the enhancement of the degradation temperature (Td) of the CS1 and CS2 scaffolds crosslinked by a higher TPP concentration. As far as the scaffold cross-linking time is concerned, the effect of this parameter on the scaffold thermal stability depended on CS concentration used for the scaffold preparation. In particular, in the case of the CS1_TPPY_z_, Td values increased when the scaffolds were crosslinked for longer times (8 h) while a slight Td reduction was observed for the ones fabricate with CS2 where presumably a pronounced effect of TPP disturbing the chain interactions was detected. As for the scaffold morphology, more homogeneous pore structures were obtained when the crosslinker concentration raised from 1 to 2% *w/v* for CS1_TPP1 at all of the experimented reaction times. In contrast, a lost in the structure homogeneity was observed when a higher CS concentration (2%) as well as higher TPP concentrations and crosslinking times were used for scaffold preparation. Generally, the scaffolds obtained with CS2 showed a more compact structure with a low pore interconnectivity (40–50%) compared to the ones obtained with CS1 (≥60%). Anyway, the crosslinking reaction improved the scaffold dimensional stability in aqueous medium and mechanical properties. Preliminary studies of biocompatibility performed on the selected scaffolds by using osteoblast cells evidenced a good cell vitality for the CS1_TPP2_8h_ sample. These results suggested how the use of a low chitosan concentration together with more severe conditions for the crosslinking reaction can favour the formation of porous structures with features suitable for application in tissue engineering.

## Figures and Tables

**Table 1:** Acronyms and properties of CS_TPP scaffolds. P = porosity; Td = degradation temperature; CM = Compressive modulus.

Sample	CS Concentration (w/v %)	TPP Concentration (w/v %)	Cross-Linking Time (h)	Td (°C)	CM (MPa)	Pore Size (µm)	Pore Regularity
CS1	1	-	-	285	0.06 ± 0.01	80–180	Good
CS2	2	-	-	290	0.2 ± 0.05	130–250	Good
CS1_TPP12h	1	1	2	255	0.2 ± 0.05	50–200	Low
CS1_TPP18h	1	1	8	262	0.5 ± 0.05	50–180	Low
CS1_TPP22h	1	2	2	270	0.6 ± 0.05	80–100	Good
CS1_TPP28h	1	2	8	277	1.2 ± 0.05	60–100	Good
CS2_TPP12h	2	1	2	262	0.3 ± 0.05	70–100	Good
CS2_TPP18h	2	1	8	247	0.9 ± 0.05	90–110	Good
CS2_TPP22h	2	2	2	282	1.8 ± 0.05	100–120	Good
CS2_TPP28h	2	2	8	273	4.7 ± 0.05	200–300	Low

**Table 2:** Intensity ratios of the absorbance at 1560 cm^−1^ (NH_2_) and 881 cm^−1^ (P-O-P) versus the absorbance at 1650 cm^−1^ (C = O) for CS_TPP samples.

Sample	A_NH2_/A_C = O_	A_P-O-P_/A_C = O_
CS1	1.96	0.58
CS2	2.02	0.58
CS1_TPP12h	1.29	0.89
CS1_TPP18h	1.10	1.03
CS1_TPP22h	1.15	1.19
CS1_TPP28h	0.94	1.50
CS2_TPP12h	1.31	0.67
CS2_TPP18h	1.28	1.04
CS2_TPP22h	1.29	1.30
CS2_TPP28h	1.00	1.44
